# Laparoscopic resection of an intramural pregnancy under transvaginal ultrasound guidance: A case report

**DOI:** 10.1097/MD.0000000000044938

**Published:** 2025-10-31

**Authors:** Shiyu Liu, Furong Tang, Xia Yin, Jiaying Ruan

**Affiliations:** a Department of Obstetrics and Gynecology, West China Second Hospital, Sichuan University, Chengdu, P.R. China; b Key Laboratory of Birth defects and Related Diseases of Women and Children, Ministry of Education, Sichuan University, Chengdu, P.R. China.

**Keywords:** diagnostic challenge, ectopic pregnancy, intramural pregnancy, minimally invasive surgery, transvaginal ultrasound

## Abstract

**Rationale:**

Intramural pregnancy is a rare form of ectopic pregnancy in which the gestational sac is embedded entirely within the myometrium, with no connection to the uterine cavity. Early diagnosis and timely management are challenging.

**Patient concerns:**

A 35-year-old Chinese woman presented with persistent abnormal uterine bleeding for 4 months following an induced abortion for a presumed embryonic arrest. Initial management at a local clinic included dilation and curettage, with no pathological villi identified, and a course of oral mifepristone. However, her serum beta-human chorionic gonadotropin levels exhibited intermittent elevation.

**Diagnoses:**

A case of intramural pregnancy was identified, in which intraoperative transvaginal ultrasonography (TVUS) enabled real-time diagnosis and facilitated precise, minimally invasive surgical management.

**Interventions:**

Subsequent TVUS at our center revealed a 1.5 × 1.1-cm heteroechoic lesion within the myometrium adjacent to the right uterine fundus and pelvic computed tomography confirmed a myometrial lesion. Hysteroscopy revealed an empty uterine cavity, which ruled out retained products of conception. The retained intramural mass was completely resected laparoscopically via hysteroscopy under real-time TVUS guidance, which enabled precise localization and preservation of healthy myometrium. Histopathological analysis confirmed the diagnosis of intramural pregnancy.

**Outcomes:**

The patient’s postoperative course was uneventful. Serum beta-human chorionic gonadotropin levels dropped to 11.4 mIU/mL on postoperative day 1 and became undetectable within 1 week. Menstrual function resumed 6 weeks after surgery. At the 3-month follow-up, the patient remained asymptomatic, with no pelvic pain or abnormal bleeding.

**Lessons:**

In cases where the lesion’s location remains unclear during laparoscopy, TVUS can assist in accurate localization and enable complete resection of the lesion.

## 1. Introduction

Intramural pregnancy is a rare subtype of ectopic pregnancy characterized by a gestational sac embedded within the myometrial muscle layer, without any connection to the uterine cavity.^[1]^ It accounts for fewer than 1% of all ectopic pregnancies, with approximately 80 cases reported in the literature to date.^[2,3]^ Early recognition and accurate diagnosis are challenging, as patients often present with nonspecific symptoms, and imaging findings may be misinterpreted as more common conditions such as retained products of conception (RPOC), adenomyosis, or fibroids. Misdiagnosis can delay appropriate treatment and increase the risk of potentially life-threatening complications, including uterine rupture and hemorrhage. Several predisposing factors for intramural pregnancy have been identified. A history of uterine surgery or trauma (such as dilatation and curettage [D&C], myomectomy, or cesarean delivery) has been reported in the majority of cases.^[4–6]^ In the largest case series, 78% of patients underwent a prior uterine surgical procedure, with D&C being the most frequently cited risk factor. First described in 1913, intramural pregnancy still lacks a clearly defined standard of care. Reported treatments options range from systemic or local administration of methotrexate to surgical excision of the ectopic tissue. Historically, many cases were managed via open surgery or even hysterectomy to control the risk of hemorrhage. However, in recent years, fertility-sparing and minimally invasive approaches have been favored when feasible. In this study, we present the case of a 35-year-old woman with an intramural pregnancy that was successfully managed with transvaginal ultrasound (TVUS)-guided laparoscopic surgery. This case report is presented in accordance with the CARE guidelines^[7]^ and highlight a novel intraoperative imaging-assisted approach that may be considered in similar scenarios to achieve precise excision while minimizing invasiveness.

## 2. Case presentation

### 2.1. Patient information

A 35-year-old Chinese woman, gravida 4, para 2, presented with a 4-month history of abnormal uterine bleeding following an induced abortion. Four months prior, at 44 days of gestation, she sought medical evaluation. Her serum beta-human chorionic gonadotropin (β-hCG) level exceeded 15,000 mIU/mL, and ultrasound revealed a deformed gestational sac measuring approximately 3.7 × 1.5 cm within the uterine cavity. The sac contained yolk sac echoes and a small amount of embryonic tissue, with no signs of cardiac tube pulsation. Her medical history was notable only for 2 prior artificial abortions. Family history was unremarkable, with no known genetic or coagulation disorders and no significant gynecological diseases among relatives. She resided in a rural area and worked as a farmer. The patient was initially diagnosed with embryonic arrest and underwent an induced abortion. Unfortunately, a pathological examination of the pregnancy tissue was not performed.

Persistent vaginal bleeding continued for approximately 4 months after the abortion. Serial TVUS scans revealed a slightly echogenic lesion measuring 2.4 × 2 cm near the anterior wall of the right uterine horn, suggestive of residual pregnancy tissue within the uterine cavity. Over the 4-month period, the patient underwent D&C, but no pathological evaluation was performed due to limited resources at the local medical facility. Following D&C, her serum β-hCG level was measured to be 6531 mIU/mL. She was subsequently started on mifepristone therapy, which gradually reduced her serum β-hCG level to 52 mIU/mL. During this period, her spouse used condoms for contraception. Despite this, vaginal bleeding persisted, and the patient was admitted to our hospital due to considerable anxiety.

### 2.2. Clinical findings

**Figure 1.**
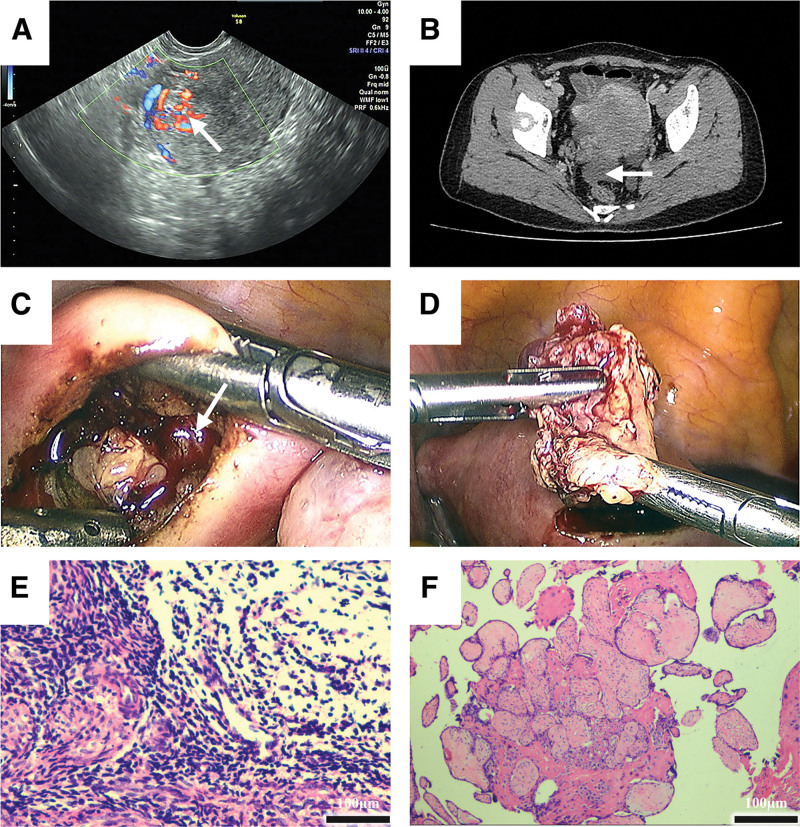
Multimodal imaging and intraoperative findings. (A) Transvaginal ultrasound image of the uterus showing an intramural lesion (arrow in (A)) in the right anterior fundal wall, measuring approximately 1.9 × 1.9 × 2.0 cm. The lesion is observed between the myometrial layers with an indistinct boundary and abundant blood flow on Doppler, indicative of trophoblastic tissue. (B) Pelvic computed tomography scan (axial view) highlighting an irregular, ill-defined density (arrow in (B)) within the right anterior uterine wall, measuring approximately 2.5 × 2.5 cm in size. The uterus is slightly enlarged. On contrast enhancement, the lesion shows mild heterogeneous uptake. (C) After localizing the site of the lesion by transvaginal ultrasound, we found soft yellow-tan lesions measuring 2 × 1 cm in the myometrium (arrow in (C)). (D) The lesion was removed without fragmentation. (E) The tissue from the uterine cavity is smooth muscle tissue, which is attached to the endometrial stroma (HE × 40 obj). Scale bar = 100 µm; H&E stain. (F) The lesion in the uterine wall was confirmed to be placental chorionic tissue (HE × 20 obj). Scale bar = 100 µm; H&E stain. H&E = hematoxylin and eosin.

On admission, her vital signs were stable. Physical examination revealed no abdominal distension or peritoneal signs. Bimanual pelvic examination demonstrated a slightly enlarged, anteverted uterus with mild tenderness on uterine palpation without adnexal masses. Speculum examination revealed a normal cervix with an open os and a small amount of dark blood in the vaginal canal. No significant cervical motion tenderness was noted. Her serum β-hCG level subsequently increased to 72 mIU/mL. TVUS revealed a lesion in the right anterior wall near the uterine fundus, located within the muscular layers (Fig. 1A). To further delineate the anatomy, pelvic computed tomography was performed, which confirmed the intramural location of the lesion in the anterior uterine wall (Fig. 1B). Although magnetic resonance imaging (MRI) is often recommended in diagnostically uncertain cases due to superior soft-tissue contrast, it was not performed in this instance due to limited immediate availability and the clarity of the ultrasound and computed tomography findings. Routine laboratory tests (including complete blood count and C-reactive protein) revealed no evidence of infection or significant anemia, thereby ruling out endometritis or coagulopathy as causes of the bleeding.

### 2.3. Diagnostic assessment

TVUS examination at the local clinic consistently demonstrated a persistent echogenic mass within the uterine wall, leading to a working diagnosis of RPOC and prompting a second D&C. However, the absence of villi on pathological examination and only a partial reduction in serum β-hCG levels introduced diagnostic uncertainty. Differential diagnoses included gestational trophoblastic disease, unusual myometrial tumors, and arteriovenous malformations. The plateauing of β-hCG at low levels over several weeks was atypical for active trophoblastic neoplasia, and the absence of systemic signs of malignancy further reduced the likelihood of a gestational trophoblastic tumor. Although a uterine arteriovenous malformation may follow D&C and cause bleeding, it is generally associated with negative β-hCG levels. The rebound increase in β-hCG from 52 to 72 mIU/mL prior to admission suggested persistent gestational tissue. Given the myometrial location of the lesion and the history of uterine trauma, an intramural ectopic pregnancy became a diagnostic consideration, despite its rarity. The patient underwent hysteroscopy under general anesthesia to exclude residual pregnancy tissue or gestational implantation. The patient was placed in the dorsal lithotomy position under standard intraoperative monitoring. Hysteroscopy revealed no pregnancy-related tissue in the uterine cavity. Intraoperative abdominal ultrasound identified a slightly echogenic area measuring 1.5 × 1.1 × 1.4 cm near the right uterine muscular wall, with indistinct borders.

### 2.4. Therapeutic intervention

We planned a minimally invasive surgical approach to remove the intramural ectopic tissue while preserving the uterus. Her husband was counseled regarding treatment options, including more aggressive surgery (hysterectomy) versus conservative management. Considering the patient’s desire for future fertility and the relatively small lesion size, conservative surgical management was selected. Although systemic methotrexate therapy was considered an alternative in similar cases, the β-hCG levels of the patient were already very low, yet demonstrated an upward trend despite prior medical management. Surgical removal was, therefore, undertaken to confirm the diagnosis and promptly resolve the condition. Mifepristone had been administered earlier at the local clinic instead of methotrexate, likely due to the declining β-hCG levels at the time and its utility in terminating early ectopic pregnancies in certain contexts.^[8]^ Furthermore, mifepristone is orally administered and readily available, making it a convenient option. The patient subsequently underwent laparoscopy for definitive diagnosis. Laparoscopic exploration was performed using 3 trocars (a 10-mm umbilical port and 2 5-mm ancillary ports). The uterus appeared normal in size and shape externally, with no visible bulging. The ovaries and fallopian tubes were unremarkable, and no significant pelvic adhesions were observed. Under laparoscopic visualization, an assistant introduced a TVUS probe. Real-time ultrasound guidance was used to precisely localize the lesion within the myometrium. This step was crucial, as the serosal surface of the uterus appeared largely normal without a bulge, and ultrasonography allowed accurate delineation of the embedded mass margins. Once localized, a 2-cm longitudinal uterine incision was made in the serosa over the guided area using a laparoscopic cautery hook, taking care to avoid deep penetration initially. The myometrium was dissected to expose and deliver the lesion. The mass, measuring approximately 2 × 1 cm in size, was yellow-tan in color and had a relatively soft consistency, consistent with the gestational tissue (Fig. 1C). It was removed en bloc without fragmentation (Fig. 1D). The base of the implantation site was curetted and lightly cauterized to ensure the complete removal of residual villi. Estimated intraoperative blood loss was 50 mL, because the lesion had small vasculature that was effectively controlled with bipolar cautery. No active bleeding was observed following resection. The uterine incision was closed in 2 layers with 2–0 absorbable sutures, achieving excellent hemostasis and restoration of uterine wall integrity. The abdomen cavity was irrigated and suctioned clear. The total operative time was approximately 1 hour, and the patient remained hemodynamically stable. After resection, the patient received standard intraoperative care, including intravenous oxytocin, to promote uterine contractions. Postoperatively, the patient was monitored for recovery and then transferred to the gynecology ward. She experienced immediate relief of symptoms, as vaginal bleeding ceased after surgery. Pathological examination confirmed the diagnosis of uterine intramural pregnancy (Fig. 1E and F). Postoperative analgesia consisted of nonsteroidal anti-inflammatory drugs and acetaminophen as needed, and the patient reported only mild pain (2/10) on the first day. Given her low risk, thromboprophylaxis was administered.

### 2.5. Follow-up and outcomes

**Figure 2.**
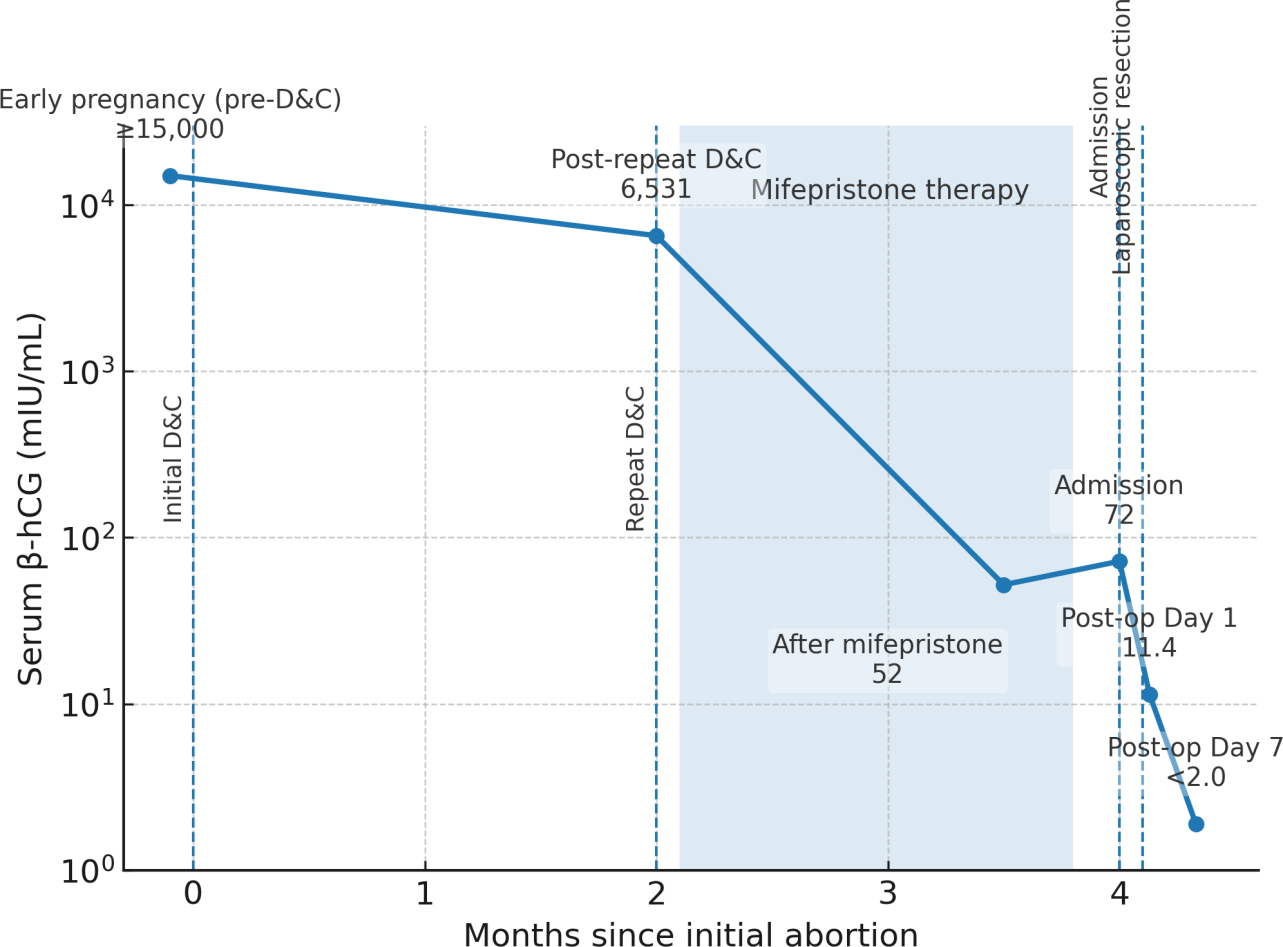
Trend of the patient’s serum β-hCG levels over time (logarithmic scale). β-hCG = beta-human chorionic gonadotropin.

The patient’s serum β-hCG level dropped to 11.4 mIU/mL on the 1st day and decreased to <2.0 mIU/mL within 1 week. She was discharged on postoperative day 3 with instructions to avoid heavy activity or intercourse for 6 weeks and to return for follow-up for β-hCG testing and ultrasound. A β-hCG trend chart was plotted over time to illustrate the course (Fig. 2). At 3 months postoperatively, the patient reported 2 normal menstrual periods, denied pelvic pain, and had an unremarkable pelvic examination. Repeat TVUS at that time was also normal. A detailed timeline of the patient’s clinical course, diagnostic procedures, and treatments is provided for clarity (Table 1). The patient expressed a high level of satisfaction with the outcome, noting complete resolution of her symptoms and a return to normal daily activities. Furthermore, she was counseled regarding fertility and obstetric implications: given the uterine incision, conception should be delayed for at least 6 months, and any future pregnancy should be closely monitored for scar integrity, consistent with post-myomectomy management protocols.

**Table 1:** Timeline of key clinical events and interventions in this case.

Time (relative to initial pregnancy termination)	Clinical event and intervention
6 wk of gestation (day 0)	Ultrasound diagnosis of embryonic demise (missed abortion) at 6 weeks. Suction D&C performed to terminate the pregnancy (the tissue was not sent for pathology).
0–1 mo post-abortion	Persistent irregular vaginal bleeding continues. Follow-up transvaginal ultrasounds show an echogenic lesion measuring ~2.4 × 2.0 cm in the right anterior uterine wall, suspicious for RPOC.
1–2 mo post-abortion	Patient underwent repeat D&C at the local hospital for suspected RPOC (no villous tissue identified pathologically due to the lack of histology facilities). The serum β-hCG after this procedure was 6531 mIU/mL, indicating persistent trophoblastic tissue.
2–4 mo post-abortion	The patient received oral mifepristone (25 mg daily) over several weeks to treat the presumed residual pregnancy tissue. Serum β-hCG levels gradually declined to 52 mIU/mL. However, vaginal bleeding persisted throughout this period.
4 mo post-abortion	Persistent bleeding and rising β-hCG levels (72 mIU/mL) prompted referral. Transvaginal ultrasound at our center showed a heterogeneous mass measuring 1.5 × 1.1 × 1.4 cm in the right anterior myometrium near the fundus (distinct from the uterine cavity) with an indistinct border and internal blood flow (Fig. 1A). Pelvic CT scan confirmed an irregular lesion in the anterior uterine wall measuring ~2.5 cm in size (Fig. 1B). Differential diagnosis included intramural ectopic pregnancy, retained gestational tissue, or a trophoblastic tumor.
Operative day	Hysteroscopy showed no abnormal tissue in the uterine cavity, effectively ruling out intrauterine RPOC or gestational trophoblastic neoplasia. Laparoscopic surgery was performed. Intraoperative transvaginal ultrasound probe was used concurrently to localize the lesion. A 2-cm purplish subserosal bulge was observed on the right anterior uterine fundus. A 2–3-cm uterine incision was made over the bulge using electrocautery, and a soft yellow mass was excised intact from within the myometrium (Fig. 1C and D). The uterine defect was sutured in 2 layers laparoscopically. Estimated blood loss was estimated to be 50 mL, and the procedural duration was 60 min. No intraoperative complications were noted.
Postoperative day 1 Postoperative day 7	The patient’s recovery was uneventful. Minimal abdominal pain (2/10 on the pain scale) was controlled with oral NSAIDs. Serum β-hCG levels dropped to 11.4 mIU/mL and hemoglobin levels remained stable. Serum β-hCG levels declined to < 2.0 mIU/mL (negative). Histopathology of the excised lesion confirmed intramural ectopic pregnancy: chorionic villi and trophoblasts were embedded within the myometrial tissue (Fig. 1F). Endometrial curettage specimen from the uterine cavity contained only decidua and smooth muscle with scant endometrial stroma, without villi (Fig. 1E).
3 mo post-surgery	The patient’s menstrual period resumed 6 wk after surgery and were reported as normal. At a 3-mo follow-up visit, she reported no pelvic pain or abnormal bleeding. Pelvic ultrasound showed a well-healed uterine scar with no residual lesion.

β-hCG = beta-human chorionic gonadotropin; CT = computed tomography; D&C = dilation and curettage; NSAIDs = nonsteroidal anti-inflammatory drugs; RPOC = retained products of conception.

Ethical approval was obtained from the Ethics Committee of the West China Second University Hospital. Written informed consent was obtained from the patient and her family for the publication of this case report and any accompanying images.

## 3. Discussion

This case report highlights several important aspects of intramural pregnancy, including diagnostic challenges, therapeutic decision-making, and the potential for fertility-sparing management through innovative techniques. To the best of our knowledge, fewer than 100 cases of intramural ectopic pregnancy have been documented, and each report contributes valuable insights into the understanding of this condition. Our case is notable for the use of intraoperative TVUS guidance during laparoscopy, which enabled precise localization and complete excision of the ectopic tissue. This approach, although relatively novel, was critical for our patient due to the small lesion size and the lack of visible external clues on the uterine surface. A recent report by Le et al similarly described the successful use of US-guided laparoscopy in managing intramural pregnancy, thereby avoiding hysterectomy.^[9]^ Such advances highlight the role of improved imaging modalities and minimally invasive techniques in enhancing outcomes for rare forms of ectopic pregnancy.

### 3.1. Diagnostic considerations

Intramural pregnancy often presents with nonspecific symptoms (abnormal bleeding and pain) and is often misdiagnosed.^[10]^ In our patient, the condition was initially mistaken for an intrauterine pregnancy failure and later diagnosed as RPOC: a common diagnostic pitfall. The literature indicates that intramural pregnancies are frequently misinterpreted as RPOC or fibroids on ultrasound. Bannon et al reported a strikingly similar case to ours: a young woman presumed to have a missed miscarriage underwent D&C, after which her β-hCG levels declined but subsequently plateaued. Imaging revealed a persistent mass that was ultimately diagnosed as an intramural ectopic pregnancy during laparoscopic surgery.^[11]^ In both Bannon case and ours, an initial post-abortion drop in β-hCG levels provided false reassurance. We, similar to Bannon et al, hypothesize that uterine curettage may partially disrupt an intramural gestation, resulting in temporary β-hCG decline and bleeding without complete removal. This scenario can mimic the expected course of a completed miscarriage, thereby delaying the correct diagnosis. Therefore, clinicians should maintain a high index of suspicion for intramural pregnancy in patients with atypical post-abortion courses (particularly when the β-hCG level plateaus or a myometrial mass persists on imaging). The early use of MRI or 3D ultrasound may improve diagnostic accuracy in such cases.

### 3.2. Management strategies

There is no universally accepted treatment for an intramural pregnancy, and management should be individualized. Conservative medical management with methotrexate has been reported, particularly for early or small intramural pregnancies. Methotrexate can be administered systemically or via direct injection into the gestational site. In 1 published case, localized in situ methotrexate injection was used to successfully treat an intramyometrial pregnancy while preserving the uterus. However, medical therapy often requires prolonged monitoring over several weeks and carries the risk of hemorrhage if the pregnancy continues to grow.^[1,2,10]^ In our patient, the use of mifepristone (an agent with a different mechanism of action, blocking progesterone support) did not completely resolve the pregnancy, although it may have limited further growth. Surgical intervention is indicated when medical therapy fails or when diagnostic uncertainty remains. Historically, many intramural pregnancies were managed with hysterectomy due to concerns about uterine rupture and uncontrollable bleeding. More recently, fertility-sparing surgical approaches have been successfully employed. Shen et al reviewed 8 cases of intramural pregnancy managed with minimally invasive techniques, several of which involved laparoscopic or hysteroscopic resection with uterine conservation.^[12]^ In our case, laparoscopic excision under real-time TVUS guidance proved to be an effective approach. The ultrasound guidance functioned as a “GPS,” enabling precise location of the lesion, accurate placement of the uterine incision, and minimizing the removal of the surrounding myometrium. This technique is particularly advantageous when the lesion is not grossly visible on the serosal surface, as was the case here, effectively transforming a potentially “blind” procedure into a targeted 1. We advocate considering this combined modality in the management of intramural pregnancies or other occult uterine lesions, as it allows complete resection with negligible blood loss and no injury to the surrounding uterine tissues.

In certain cases, additional strategies have been described to mitigate surgical risks. For example, temporary uterine artery occlusion or embolization can reduce perfusion in an intramural pregnancy, thereby minimizing intraoperative bleeding. This step was not required in our case, as the lesion was small and bleeding was effectively controlled with standard cautery and sutures. Nonetheless, in larger or more vascular intramural ectopic lesions, preoperative uterine artery embolization or the use of intraoperative tourniquets should be considered.^[3]^

### 3.3. Fertility implications

Preserving fertility and uterine integrity is a primary concern in treating intramural pregnancies. In our case, the patient expressed a desire for future fertility; therefore, avoiding hysterectomy was paramount. A minimally invasive approach allowed for uterine preservation. We recommended an adequate healing period before future conception and emphasized the importance of close monitoring in any subsequent pregnancy to detect potential complications (such as scar rupture if labor ensues). Successful pregnancies have been reported after conservative management of intramural ectopic pregnancies. For instance, Gilmore et al reported a case of intramyometrial pregnancy in a woman with a uterine septum. After laparoscopic excision of the ectopic and septal resection, the patient later had a normal intrauterine pregnancy.^[10]^ Similarly, other case reports noted preserved fertility and live births following the treatment of intramural pregnancy.^[13]^ In our case, although the patient did not attempt to conceive, successful removal of the lesion with minimal damage to the uterus should theoretically leave her fertility intact. We also provided counseling regarding the need for early US examinations in any future pregnancy to confirm proper implantation.

### 3.4. Patient outcomes and follow-up

Both the clinical follow-up and subjective feedback were used to evaluate the outcomes. At 3 and 6 months, the patient reported regular menses and no pelvic pain, indicating satisfactory gynecological recovery. We did not employ a formal standardized questionnaire for patient-reported outcomes (such as Short Form-36 or a specific quality-of-life measure) owing to the single-case context; however, qualitatively, the patient reported a significant improvement in her quality of life, high satisfaction with the treatment, and relief from avoiding a hysterectomy. In future cases, it may be beneficial to utilize validated measures (e.g., pain scores and quality-of-life scales) to quantitatively assess recovery after intramural pregnancy treatment, although no such disease-specific tool exists. As an objective outcome measure, we also plotted the serial β-hCG levels (Fig. 2). A rapid decline to 0 is an important indicator of complete resolution of ectopic pregnancy tissue.

### 3.5. Strengths and limitations

This case report adds to the growing literature on intramural pregnancy by illustrating a successful management strategy and long-term follow-up in an initial low-resource setting. The strengths of our approach include the integration of diagnostic and therapeutic modalities (combining hysteroscopy, laparoscopy, and ultrasound) to minimize uncertainty and invasiveness, as well as the benefit of multidisciplinary input from radiology, minimally invasive surgery, and pathology: which likely contributed to the favorable outcomes. One limitation is that this was a single-case report, and the results may not be generalizable to all intramural pregnancies, especially those with larger or later gestations. Additionally, our patient’s follow-up, while positive for 6 months, does not yet include an actual subsequent pregnancy outcome. Therefore, the long-term fertility results remain to be determined. Additionally, some imaging modalities (MRI) and potential laboratory tests were not utilized; in a more resource-rich scenario, these might provide additional confirmation of the diagnosis.

### 3.6. Comparison with other case reports

The management of an intramural pregnancy varies in the literature. Some reports advocate first medical management, especially if the patient is stable, whereas others favor prompt surgical intervention to prevent complications. Our case report demonstrates that, even after a trial of medical therapy (mifepristone) failed, surgical management is still effective and safe. Compared with similar case reports, the use of intraoperative US in this case is a distinguishing feature. Not all centers have reported using this strategy, although it has been suggested as helpful in at least 1 other case. We believe that this technique should be extensively reported and used in such scenarios. Moreover, this case underlines the importance of not dismissing persistent low β-hCG levels, as even a modest elevation can signify an ongoing ectopic pregnancy that warrants intervention. Primary care and rural clinicians should be aware of intramural pregnancy as a rare possibility in patients with unusual post-abortion courses. In our patient’s rural context, limited pathology and imaging resources delayed her diagnosis. Improving access to advanced US or referral to specialty centers can be lifesaving in similar situations.

## 4. Conclusion

In summary, we successfully treated a rare intramural ectopic pregnancy using fertility-sparing laparoscopic surgery aided by TVUS localization. Clinicians should remain vigilant for intramural pregnancy in patients with risk factors (such as prior uterine procedures) who present with persistent bleeding and discordant β-hCG trends. Our case supports a growing body of evidence that minimally invasive approaches, when carefully planned, can effectively manage this condition. Future contributions, including a case series and an international registry, will hopefully establish clearer guidelines. In conclusion, early recognition and individualized management are essential for intramural pregnancy. When the location of the ectopic tissue is unclear during laparoscopy, employing intraoperative ultrasound can assist in precisely locating and excising the lesion. This combined approach maximizes the chance of removing the ectopic tissue (achieving a prompt decline in β-hCG levels) while minimizing unnecessary damage to the uterus, thereby preserving the reproductive potential of the patient.

## Author contributions

**Writing – original draft:** Shiyu Liu.

**Writing – review & editing:** Furong Tang, Xia Yin, Jiaying Ruan.
